# Factors Influencing Central Nervous System Abnormalities in m.11778G>A Carriers

**DOI:** 10.3390/brainsci10080513

**Published:** 2020-08-03

**Authors:** Josef Finsterer

**Affiliations:** Klinik Landstrasse, Messerli Institute, 1180 Vienna, Austria; fipaps@yahoo.de

**Keywords:** m.3243A>G, mtDNA, mitochondrial, rhabdomyolysis, ventricular arrhythmia

With interest, we read the article by Jonak et al. about a cerebral imaging study on 15 patients with Leber’s hereditary optic neuropathy (LHON) by means of a 7T-MRI [1]. The authors found atrophy of the globus pallidus bilaterally, the right nucleus accumbens, and the optic chiasm, and subcortical white matter atrophy [1]. We have the following comments and concerns

A shortcoming of the study is that it is not reported if any of the 15 included patients received idebenone (Raxone®), the current standard therapy of LHON, at the time of the investigation. Additionally, it is conceivable that the patients received other antioxidants or cofactors that could have influenced the results. Knowing the current medication is crucial as particularly idebenone may not only lead to recovery of visual impairment but also to morphological regression of potential cerebral abnormalities on imaging. 

A further shortcoming is that it is not reported which central nervous system (CNS) abnormalities were seen in the 15 patients on conventional imaging. CNS involvement other than affection of the retinal ganglion cells has been repeatedly reported in patients with LHON and includes white matter lesions (WMLs), cerebral atrophy, optic atrophy, basal ganglia lesions [2], brainstem lesions [3], and multiple sclerosis-like plaques. WMLs are a common finding in patients with LHON [4]. They may be found even in asymptomatic carriers of LHON mutations [5]. Some of these lesions are occasionally interpreted as multiple sclerosis plaques or multiple sclerosis-like plaques [6]. We should know if any of the 15 included patients had CNS abnormalities previously reported on conventional imaging. 

A third shortcoming of the study is that heteroplasmy rates of the variant m.11778G>A were not reported. Though homoplasmic in the majority of the cases, there are also single cases in which the m.11778G>A variant is pathogenic, even in the heteroplasmic state [7]. We should know if atrophy of the globus pallidus and subcortical white matter atrophy correlated positively with the heteroplasmy rate of the m.11778G>A variant. 

From experimental studies, it is known that LHON patients may profit from application of the ketogenic diet (KD) [8]. We should know how many of the included patients adhered to the KD at the time of the study investigations. If KD is beneficial, it may strongly influence the study results. 

Missing is the family history of the included patients. We should know how many of the included patients had inherited the disease and in how many patients the mutation occurred spontaneously. The penetrance of the variant should be presented in hereditary cases.

In the abstract, it is mentioned that the volume of both “palladiums” was lower in LHON carriers compared to controls. Do the authors mean the globus pallidus?

No explanation is provided why only the right nucleus accumbens showed a volume loss. 

Overall, this interesting study has a number of shortcomings, which need to be addressed before drawing final conclusions. The authors should provide the current medication, conventional imaging results, heteroplasmy rates of the m.11778G>A variant, the family history, and if any of the patients adhered to the KD.
